# DETERMINING THE PREVALENCE OF SARCOPENIA IN AN INPATIENT GERIATRIC POPULATION USING EWGSOP2 OR FNIH DEFINITIONS

**DOI:** 10.1093/geroni/igz038.3107

**Published:** 2019-11-08

**Authors:** Matthew M Scholl, Elena Volpi, Rachel R Deer

**Affiliations:** 1 University of Texas Medical Branch, Galveston, Texas, United States; 2 UTMB, Galveston, Texas, United States

## Abstract

Sarcopenia has been recognized as a progressive and generalized skeletal muscle disorder leading to loss of strength, muscle mass, and function. It is associated with an increased likelihood of adverse outcomes like falls, fractures, physical disability, and mortality. International consensus groups continue providing new definitions and clinical cut-off points despite over a decade of work in this area. Thus, the purpose of this research was to determine the prevalence of sarcopenia using two of the most current operational definitions (Foundation of NIH Sarcopenia Project (FNIH) and the European Working Group on Sarcopenia in Older Persons 2 (EWGSOP2)). Our cohort of acutely hospitalized older adults was formed from combining data from two randomized controlled trials and one cross-sectional observational study. Testing during hospitalization included measures of: demographics, body composition (DEXA), physical function tests, psychological wellbeing and independence questionnaires, and chart review. These were used to analyze the cohort according to three main groupings of low physical performance, low muscle strength, and low lean mass. We compared multiple tests and cutoffs for each of the three groupings under the FNIH and EWGSOP2 definitions, which varied 3% for low lean mass up to 48% for tests of low physical performance. After examining the efficacy of each cutoff, we evaluated the differences between FNIH and EWGSOP2. In our cohort, the prevalence of sarcopenia was 15.79% by EWGSOP2 and 13.59% by FNIH. The groupings within FNIH and EWGSOP2 were found to be near identical across almost all measures despite the definitions’ discrepancies in cutoff points.

